# Potential impact of relational job design on future intentions of episodic volunteers in major sport events

**DOI:** 10.3389/fpsyg.2024.1302316

**Published:** 2024-05-15

**Authors:** Jingxuan Su, Haifeng Li, Hongyu Ma

**Affiliations:** ^1^School of Psychology, Central China Normal University, Wuhan, Hubei, China; ^2^School of Physics, South China Normal University, Guangzhou, Guangdong, China

**Keywords:** relational job design, organizational commitment, commitment to beneficiaries, perceived organizational support, future volunteer intentions, Social Identity Theory, questionnaire

## Abstract

**Introduction:**

Based on Social Identity Theory, this study hypothesized the parallel mediating roles of organizational commitment, and commitment to beneficiaries, in the relationship between relational job design and future volunteer intentions among episodic volunteers at a mega sport event. Perceived organizational support was tested as a moderator of this relationship.

**Methods:**

Participants were 617 episodic volunteers (35.7% male and 64.3% female) at the 7th CISM Military World Games in Wuhan, China, who completed online questionnaires.

**Results:**

Regression-based analyses indicated that relational job design positively predicted future volunteer intentions through organizational commitment. Although the results did not indicate a mediating role of commitment to beneficiaries, relational job design was still shown to positively predict commitment to beneficiaries. Furthermore, the association between relational job design and commitment to beneficiaries was moderated by perceived organizational support, such the effect was stronger when perceived organizational support was high.

**Discussion:**

The results have practical implications for strengthening episodic volunteers’ intentions to participate in future mega sport events, creating a legacy of volunteerism.

## Introduction

Volunteers contribute to the success of sport events of all types and sizes (Cuskelly et al., 2006). They may be especially helpful in major sport events that attract a large number of visitors as well as mass media attention (Munjal et al., 2021; Cnaan et al., 2022). Examples of major sport events include the Olympic Games, FIFA World Cup, and Alpine World Ski Championships (Bladen, 2008; Wollebæk et al., 2014; Kim et al., 2019; Dickson et al., 2020; Teixeira et al., 2023). Volunteers in major sport events are usually episodic volunteers (Neufeind et al., 2013) who engage in one-time or short-term volunteering (Cnaan and Handy, 2005). Compared to ongoing volunteers, episodic volunteers have been shown to be less likely to participate in another volunteer event (Hyde et al., 2016) because their tasks are rather simple, repetitive, and uneasy to reflect their impact (Yoo et al., 2023). So they have not built psychological commitment with the temporary organization or the cause they serve (Nichols, 2013; Power and Nedvetskaya, 2022).

Episodic volunteers’ future intentions, defined as their willingness to participate again in another similar event, are significant for managing volunteers. First, as major sport events recruit volunteers temporarily and in large numbers (e.g., 70,000 volunteers for the 2012 Olympic Games in London, and 20,000 volunteers for the 2022 FIFA World Cup in Qatar; Wicker, 2017), there are high costs associated with training volunteers (Snyder and Omoto, 2008; Alfes et al., 2015). If volunteers with previous experience are willing to participate again in a future event, training costs would likely be lower. Second, volunteers with previous experience have been shown to be more likely to recognize the importance of their role in making the event a success, and to perform better in interacting with fans, than new volunteers (Khoo and Engelhorn, 2011). Third, episodic volunteers’ high future intentions represent the event’s success in creating a strong legacy of volunteerism (Downward and Ralston, 2006; Doherty, 2009). People who have volunteered more than once can share their skills and values with new volunteers, perpetuating the spirit of volunteerism. Episodic volunteers’ future intentions have been shown to be associated with personality, personal experiences and management practices. Cho et al. (2023) proved extraversion and agreeableness could improve continuance volunteer intention through enhancing volunteer motivation. The volunteer’s emotional experiences while volunteering, such as high satisfaction (Neufeind et al., 2013; Hyde et al., 2014; Compion et al., 2022) and a feeling of attachment or obligation to the organizers and sponsors of the event (Cuskelly and O’Brien, 2013; Bruening et al., 2015), are associated with a willingness to participate in another event. Management practices are also associated with future intention. Rogalsky et al. (2016) found that perceived effective supervision might positively predict the future intentions of sport event volunteers by decreasing role ambiguity and increasing role satisfaction. Management practices such as maintaining good communication, recognizing achievements, and creating jobs that are rewarding could also improve volunteers’ experience and increase future intentions (Taylor et al., 2006; Stirling et al., 2011; Hyde et al., 2016; Benevene et al., 2018).

Relational job design is an important management practice that might influence episodic volunteers’ future intentions (Loghmani et al., 2024). The concept of relational job design was developed in the paid employment context and refers to a job’s structural properties that enable employees to connect with others (Grant, 2007). Relational job design can also exist in the context of volunteerism (Alfes et al., 2015). The assumption is that volunteers will invest more time and energy into tasks that allow them to see their impact on the people they serve. Commitment has been proved to be important to high-quality-volunteer behavior (Xu et al., 2020). Relational job design has been shown to be effective in improving long-term volunteers’ commitment to the organization and to the beneficiaries of the volunteer work, in decreasing turnover intentions, and in extending volunteer time (Alfes et al., 2015). It is important to test whether relational job design also benefits episodic volunteers, as ongoing volunteers and episodic volunteers differ significantly in time, motivation, and experience (Cnaan et al., 2022). Munjal et al. (2021) offered an integrated model of volunteer job design, including relational job design.

This study makes the following contributions to the literature. First, we investigate the relationship of relational job design and future intentions among episodic volunteers in a major sport event to expand evidence of the effectiveness of relational job design for episodic volunteers in addition to ongoing volunteers. We invited volunteers at the 7th CISM Military World Games to fill online questionnaires and used regression-based analyses to test this moderated mediating model. Social Identity Theory maintains that individuals tend to benefit organizations when they feel organizational-based self-esteem through recognizing themselves as a valuable member (Tajfel and Turner, 1985; Turner, 1985). While relational job design makes episodic volunteers see how they are valuable, we proposed that episodic volunteers with high relational job design will feel high self-esteem and continue to do beneficial things for voluntary like re-participate in another major sport event as a volunteer.

Second, we explore the underlying mechanism of the relationship between relational job design and episodic volunteers’ future intentions from a new perspective. According to Social Identity Theory, job design influences individuals’ desirable outcomes related to organizations by increasing commitment (Tajfel and Turner, 1985; Turner, 1985). Some researchers have asserted that episodic volunteers have low commitment to the organization because of the brief and temporary nature of the work (Cnaan et al., 2022). However, we assert that episodic volunteers, especially in mega sport events, can develop commitment. Taking China as an example, episodic volunteers of mega sport events are jointly managed by government sport departments and the University Communist Youth League Committee, which are both stable government-run volunteer organizations (Yao and Schwarz, 2017; Wu, 2018). It is possible for volunteers to form commitment to these organizations affiliated with the government. In addition, commitment has multiple foci (Valéau et al., 2013), so there are multiple ways to show commitment in the context of mega sport events. Volunteers may be committed to the organization but also to the beneficiaries of the volunteering, such as athletes who participate consistently in sport events (Hyde et al., 2016). Commitment to the organization and commitment to beneficiaries are different because they reflect an individual’s bond with different targets (Valéau et al., 2013; Xu et al., 2020). In the current study we analyze these two forms of commitment separately as parallel mediators of the association between relational job design and volunteers’ future intention.

Third, we explored a boundary condition, namely perceived organizational support, of the association between relational job design and future intentions. Organizational support is characterized by efforts to helps volunteers to avoid and handle hassles, to perform better in their work, and to experience more positive feedback (Fuller et al., 2006; Cheng et al., 2016). With organizational support, volunteers are more likely to devote themselves to and benefit from relational job design, increasing commitment to the organization and beneficiaries (Tajfel and Turner, 1985; Turner, 1985). Our results will clarify whether organizational support strengthens or weakens the association between relational job design and volunteers’ future intention.

## Theoretical background

Social Identity Theory (Tajfel and Turner, 1985; Turner, 1985) holds that individuals are motivated to maintain or enhance their self-esteem, and the tendency to identify with groups who provide them with organization-based self-esteem refers to “an individual evaluating his or her personal adequacy and worthiness as an organizational member” (Gardner and Pierce, 1998). People form commitment by acting in ways that supports their group (Ashforth and Mael, 1989; Fuller et al., 2006). Commitment is defined as a force that connects an individual to a target, and to a course of action that is pertinent to the target (Meyer et al., 2004).

In this study, we adopted Social Identity Theory (Tajfel and Turner, 1985; Turner, 1985) as a framework for examining the relationship between relational job design and future volunteer intentions. We tested a model in which this association is mediated by organizational commitment and commitment to beneficiaries, and the association is moderated by perceived organizational support. Social Identity Theory is relevant for understanding the social factors of interest in the current study, such as relational jobs, commitment to a group and members of a group, and group support.

Grant (2007) developed a conceptual model of relational job design in the workplace to elucidate ways to enhance employees’ prosocial behavior. Subsequent studies have confirmed the benefits of relational job design in fostering self-determination and encouraging helping behavior (Hernaus et al., 2021; Robertson et al., 2023). Consequently, supervisors in various fields, particularly among nurses, doctors, and teachers, are actively striving to create more relational job roles and promote relational job crafting among employees (Santos et al., 2016; Noesgaard and Jørgensen, 2023). Given that prosocial behavior is a primary focus in volunteerism research, Alfes et al. (2015) conducted a pioneering study examining the impact of relational job design on ongoing volunteers. This groundbreaking work has prompted volunteer managers to place greater emphasis on incorporating relational job design strategies. They are working diligently to facilitate closer connections between volunteers and beneficiaries, thus enhancing the volunteers’ sense of fulfillment and impact (Alfes et al., 2015; Munjal et al., 2021; Loghmani et al., 2024). In a novel approach, our study seeks to ascertain the relevance of relational job design for episodic volunteers, further contributing to the understanding of volunteer motivations and behaviors.

Perceptions of relational job design encompass “a way of experiencing one’s work as significant and purposeful through its connection to the welfare of other people” (Grant, 2007), an example of organization-based self-esteem. When relational job design is high, employees may feel significance and worthiness as a member of the volunteer organization, creating high organization-based self-esteem (Fuller et al., 2006). As a result, they will commit to the volunteer group and would like to participate in such volunteer activities again. Volunteers can also be committed to beneficiaries of their work (Valéau et al., 2013). When beneficiaries express appreciation, volunteers may want to serve them again in the next event. Moreover, based on Social Identity Theory (Tajfel and Turner, 1985; Turner, 1985), we can expect that organizational support provides volunteers with self-esteem and helps them to contribute more and benefit more from relational-job-design tasks. These contributions can improve the effectiveness of relational job design. We propose that perceived organizational support may moderate the relationship between relational job design and different foci of commitments.

## Hypothesis development

### Mediating effects of episodic volunteers’ organizational commitment and commitment to beneficiaries

Relational job design refers to a job’s structural properties that enable employees to connect with others (Grant, 2007). Grant (2007) stated that when someone’s job has relational characteristics, they will perceive themselves as having impact on others. People who benefit from their work are called beneficiaries. For instance, teachers’ beneficiaries are students, sellers’ beneficiaries are clients, and episodic volunteers’ beneficiaries are groups such as audiences, athletes, and media reporters in sport events (Grant, 2007). Because connecting with and having an impact on beneficiaries is one of the main motivations of episodic volunteers (Khoo and Engelhorn, 2011), relational job design should increase their motivation, and enhance their identification as a volunteer and a member of the volunteer organization (Traeger and Alfes, 2019). Also, episodic volunteers might attribute relational job design to their organization’s respect for them (Turner and Reynolds, 2001). Moreover, relational job design reflects the organization’s values by communicating the idea that it is important for volunteers to benefit others (Alfes et al., 2015). This value is shared by most episodic volunteers (Cnaan et al., 2022). The consistency of these values facilitates volunteers’ identification with the organization (Finegan, 2000).

Alfes et al.’ (2015) study showed that relational job design improved ongoing volunteers’ organizational commitment. Other researchers obtained similar findings in work settings. A study of nurses documented that relational job characteristics explained affective commitment to the hospital through nurses’ work engagement (Santos et al., 2016). Asuah-Duodu et al. (2019) reported that physicians’ relational job design was positively associated with their organizational commitment. Robertson et al. (2023) proved that relational job design enhanced prosocial motivation of employees both on the frontline and non-frontline during COVID-19. Thus, we hypothesized:

*Hypothesis 1a*: Relational job design will be positively associated with episodic volunteers’ organizational commitment.

Commitment has multiple foci (Reichers, 1985; Becker, 1992). In addition to organizational commitment, volunteers may form commitment to beneficiaries (Alfes et al., 2015). There are at least two reasons for this commitment. On the one hand, episodic volunteers will receive expressions of appreciation from beneficiaries, building their self-esteem as a member of the volunteer group (Turner and Reynolds, 2001) and of the organization (Mael and Ashforth, 1992; Fuller et al., 2006). On the other hand, episodic volunteers will form emotional bonds with beneficiaries with whom they have frequent contact (Turner and Reynolds, 2001), showing a form of emotional commitment. Thus, we hypothesized:

*Hypothesis 1b*: Relational job design will be positively associated with episodic volunteers’ commitment to beneficiaries.

There is also appears to be a connection between episodic volunteers’ commitment and their future intensions. Hyde et al. (2016) showed that organizational commitment predicted episodic volunteers’ intentions to continue volunteering. Another study conducted among volunteers in biking race events indicated that organizational commitment predicted intentions to remain as a volunteer for future events (Mykletun and Himanen, 2016). We propose that episodic volunteers’ organizational commitment will be directly associated with their future volunteer intentions for the following three reasons. First, when episodic volunteers are highly committed to volunteer organizations, they may feel more involved in the organizational activities and be more likely to achieve organizational goals (Rhoades et al., 2001). These experiences may lead them to form ongoing intentions to volunteer, a kind of extension of involvement. Second, affective commitment encourages volunteers to develop proactive attitudes toward volunteerism and to value the specific tasks of the volunteer organization more highly (Meyer and Allen, 1997). Volunteers who show more affective commitment may also show more willingness to exert extra effort to stay with the volunteer organization. Third, high organizational commitment increases volunteers’ desire to maintain their role as a volunteer and the likelihood that they will volunteer again (Luchak and Gellatly, 2007).

Thus, we hypothesized:

*Hypothesis 2a*: Episodic volunteers’ organizational commitment will positively predict their future volunteer intentions.

Social Identity Theory predicts that people will support the groups to which they are committed (e.g., Ashforth and Mael, 1989; Mael and Ashforth, 1992). When episodic volunteers commit to beneficiaries, they may develop a sense of liking them and wishing to serve their best interests (Valéau et al., 2013). The episodic volunteers may then be more likely to help these beneficiaries again if there is another chance to do so. In addition, volunteers who form high commitment to beneficiaries have a need for attachment and relatedness to the beneficiaries (Meyer et al., 2004). Volunteers can continue to satisfy their need for relatedness by volunteering in future events (Deci and Ryan, 1985).

Although this line of reasoning is relevant to the current study, there is a lack of evidence that commitment to beneficiaries predicts episodic volunteers’ future intentions. However, research conducted among ongoing volunteers has shown that commitment to beneficiaries was positively related to positive outcomes like time spent volunteering (Alfes et al., 2015) and negatively related to negative outcomes like turnover intentions (Valéau et al., 2013; McAllum, 2017). Thus, we hypothesized:

*Hypothesis 2b*: Episodic volunteer commitment to beneficiaries will positively predict their future volunteer intentions.

Based on Social Identity Theory (Tajfel and Turner, 1985; Turner, 1985), it can be expected that commitment would mediate the association between factors that provide self-esteem and individuals’ behaviors that support their groups. For instance, Alfes et al. (2015) demonstrated that organizational commitment and commitment to beneficiaries both mediated the association between relational job design and ongoing volunteers’ turnover intention. Other results also demonstrated a mediating role of commitment (Asuah-Duodu et al., 2019; Ribeiro et al., 2020). Thus, we hypothesized:

*Hypothesis 3a*: Episodic volunteer organizational commitment will mediate the relationship between relational job design and future volunteer intentions.

*Hypothesis 3b*: Episodic volunteer commitment to beneficiaries will mediate the relationship between relational job design and future volunteer intentions.

### Perceived organizational support as a moderator

Perceived organizational support is defined as the extent to which employees believe that their organization values their contributions and cares about their well-being (Eisenberger et al., 1986). Individuals who receive more organizational support are more satisfied with the organization (Eisenberger et al., 1997) and think more highly of the volunteer organization (Prayag et al., 2020). Thus, they may attribute relational job design to the organization’s respect, and forming organizational commitment. Moreover, because organizational support provides job resources and helps volunteers perform better (Chen et al., 2020), volunteers might develop a stronger identification as a volunteer through relational job design. Previous studies that recruited paid employees as participants suggested that perceived organizational support could reinforce the positive relationship between job redesign and organizational commitment or positive outcomes resulting from commitment (Cheng et al., 2016; Cheng and Yi, 2018; Chang et al., 2020). Thus, we hypothesized:

*Hypothesis 4a*: Perceived organizational support will moderate the relationship between relational job design and episodic volunteer organizational commitment, such that the relationship will be stronger when perceived organizational support is high.

We also assume that organizational support provides a favorable environment for episodic volunteers to focus on relational job design and form a commitment to beneficiaries. To be specific, organizational support provides resources for employees to deal with job demands (Bakker and Demerouti, 2017) when contacting and working for beneficiaries. As a result, they may be more likely to perform better with relational job design (Imran and Aldaas, 2020) and be more appreciated by those beneficiaries. Moreover, compared to volunteers with lower perceived organizational support, those with high support might have more positive affect and experiences with beneficiaries, which are essential for forming affective commitment (Wicker, 2017). There is empirical support for this view. Cheng et al. (2016) found that perceived organizational support moderated, strengthening the relationship between tour leaders’ job redesign and organizational commitment. Thus, we hypothesized:

*Hypothesis 4b*: Perceived organizational support will moderate the relationship between relational job design and episodic volunteer commitment to beneficiaries, such that the relationship will be stronger when perceived organizational support is high.

**Figure 1:**
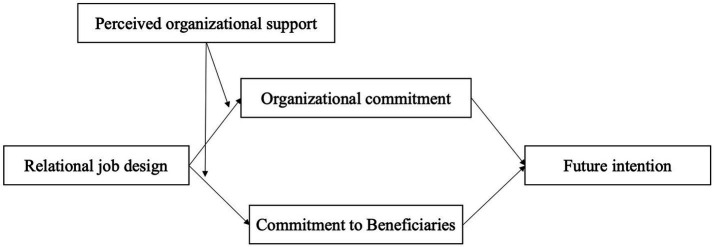
Overview of the hypothesized moderated mediation model.

Previous combined mediation and moderation models in other fields (Muller et al., 2005; Hayes, 2013) can inform our predictions about our own conceptual model. We started with the idea that organizational commitment and commitment to beneficiaries will mediate the relationship between relational job design and future volunteer intentions. We then added the idea that perceived organizational support moderates, strengthening the relationship between relational job design and commitment (organizational commitment and commitment to beneficiaries). The combined analyses constitute a moderated mediation model that examines the roles of organizational commitment, commitment to beneficiaries, and perceived organizational support in relation to the association between relational job design and future volunteer intentions. The moderated mediation model is shown in Figure 1. Thus, we hypothesized:

*Hypothesis 5a*: Perceived organizational support will moderate the indirect relationship between relational job design and future volunteer intentions through episodic volunteers’ organizational commitment, such that the indirect relationship will be stronger when perceived organizational support is high.

*Hypothesis 5b*: Perceived organizational support will moderate the indirect relationship between relational job design and future volunteer intentions through episodic volunteers’ commitment to beneficiaries, such that the indirect relationship will be stronger when perceived organizational support is high.

## Method

### Participants and procedure

The participants were Chinese adults who were volunteers at the 7th CISM Military World Games in Wuhan, China. Data collection occurred a week after the event finished. We contacted the supervisors of these volunteers to help invite volunteers to join in our survey. The participants read the instructions, clarified the purpose of the scales, and explained that responses to the questionnaires were anonymous. They completed the online questionnaires within 10 min according to their actual volunteer experience. Participation was voluntary, and participants received a gift worth ¥20 (approximately US$3) at the end of the data collection. The study received approval from the research ethics committee at the university with which the authors who conducted data collection were affiliated.

A total of 750 volunteers completed the questionnaires. After we excluded questionnaires that appeared invalid (more than 33% of the variables were missing, or participants gave the same response on more than 90% of the items), we had a final sample of 617 volunteers. All participants in the final sample were college students. Women accounted for 64.3% of the sample. Approximately 92.2% of respondents were aged 18–25, 5.1% were older than 25, and 2.6% were younger than 18.

### Measures

The following English-language scales were translated into Chinese using the translation and back-translation method (Brislin, 1980).

#### Relational job design

To measure relational job design, we used a 4-item scale adapted from Grant (2007) to suit the volunteering context. Participants rated items on a 7-point Likert scale from 1 (“strongly disagree”) to 7 (“strongly agree”). An example item was “Through my volunteering work, I substantially improve the welfare of [the organization’s] beneficiaries.” The Cronbach’s alpha was 0.92.

#### Organizational commitment

To measure affective commitment to the volunteer organization, we used an 8-item scale (Allen and Meyer, 1990) that was translated by Chen et al. (2006) for Chinese respondents. We adapted the scale to suit the volunteering context. Participants rated items on a 6-point Likert scale from 1 (“strongly disagree”) to 6 (“strongly agree”). An example item was “I feel a strong sense of belonging to the volunteer organization.” The Cronbach’s alpha was 0.89.

#### Commitment to beneficiaries

To measure commitment to beneficiaries, we changed the wording in a 6-item measure by Grant (2007) to suit the volunteering context. We also dropped the two reverse scored items in the original scale. Items with reverse scoring may affect the scale reliability due to the expression effect (Marsh, 1986), resulting in cognitive speed impairment in the response process. Participants rated the remaining four items on a 5-point Likert scale from 1 (“strongly disagree”) to 5 (“strongly agree”). An example item was “I am strongly committed to the beneficiaries of my volunteering activities.” The Cronbach’s alpha was 0.87.

#### Future volunteer intentions

To measure volunteers’ future intentions, we used the 3-item Purchase Intentions Scale (Maxham, 2001) in an adapted version designed to suit the volunteering context (Lee et al., 2016). Participants rated items on a 7-point Likert scale from 1 (“strongly disagree”) to 7 (“strongly agree”). An example item was “I plan to volunteer for a similar event like 7th CISM Military World Games again.” The Cronbach’s alpha was 0.93.

#### Perceived organizational support

To measure perceived organizational support, we used a 4-item scale adapted from Boezeman and Ellemers (2007) to suit the volunteering context. Participants rated items on a 7-point Likert scale from 1 (“strongly disagree”) to 7 (“strongly agree”). An example item was “The volunteer organization of the 7th CISM Military World Games makes volunteers recognize their effort’s worth.” The Cronbach’s alpha was 0.92.

#### Control variables

According to prior research on volunteer attitude and behavior (Wilson, 2012), we chose gender (male = 1; female = 0), age, education (master’s degree or above = 2; bachelor’s degree = 1; junior college degree and below = 0) as control variables.

### Statistical analysis

To test our hypotheses through linear regression-based analysis, we used the SPSS macro PROCESS (Hayes, 2013), which is used to test complex models including both mediator and moderator variables. We used PROCESS Model 4 to test the parallel mediating effects of organizational commitment and commitment to beneficiaries in the relationship between relational job design and future volunteer intentions (Hypotheses 1a, 1b, 2a, 2b, 3a, and 3b). We used PROCESS Model 7 to test the moderated mediation effect (Hypotheses 4a, 4b, 5a, and 5b). We used PROCESS Model 1 to generate the output for probing and graphing interactions. In the present study, bootstrapped bias-corrected confidence intervals (95%) for the indirect effects were generated using 5,000 iterations of bootstrapping. An effect is considered significant if the confidence interval does not include 0.

## Results

### Normality test

We used Kolmogorov Smirnov method to test the normality of each variable. The significance values of relational job design, perceived organizational support, organizational commitment, commitment to beneficiaries, future intentions were, respectively, 0.49, 0.62, 0.36, 0.44, and 0.27 (*p* > 0.05), meaning that each data was normally distributed so that it can be concluded that each variable had a distribution of normally distributed data.

### Linearity test

We did the linearity test and the results indicated linearity or the presence of a straight line that connects relational job design and organizational commitment (*F* = 18.06, *p* < 0.01), relational job design and commitment to beneficiaries (*F* = 15.06, *p* < 0.01), organizational commitment and future intentions (*F* = 18.15, *p* < 0.01), commitment to beneficiaries and future intentions (*F* = 10.27, *p* < 0.01), relational job design and future intention (*F* = 12.51, *p* < 0.01).

### Preliminary analyses

We used Harman’s single factor test in SPSS to detect common method variance bias in our data. When all items were converged onto a single unrotated factor, the single factor explained over 45.28% of the total variance, which is less than the 50% threshold value (Chaubey et al., 2019). The results showed that common method variance did not substantially bias the results in this study.

### Descriptive statistics and correlations

Table 1 shows the means, standard deviations, and correlations among all study variables. Relational job design was positively related to organizational commitment (*r* = 0.59, *p* < 0.001), commitment to beneficiaries (*r* = 0.52, *p* < 0.001), and future volunteer intentions (*r* = 0.45, *p* < 0.001). Additionally, organizational commitment and commitment to beneficiaries was positively related to future volunteer intentions (*r* = 0.59, *p* < 0.001; *r* = 0.38, *p* < 0.001). These results provided preliminary support for our hypotheses.

**Table 1:** Means, standard deviations, and correlations among study variables.

Variable	*M*	*SD*	1	2	3	4	5	6	7	8
1. Gender	—	—	—							
2. Age	—	—	−0.08	—						
3. Education background	—	—	−0.13	0.17^**^	—					
4. Relational job design	6.60	0.98	−0.04	0.04	−0.11^**^	—				
5. Perceived organizational support	6.27	1.03	0.07	0.04	−0.14^**^	0.71^***^	—			
6. Organizational commitment	5.13	0.79	0.02	0.05	−0.05	0.59^***^	0.70^***^	—		
7. Commitment to beneficiaries	4.18	0.75	−0.01	0.08	−0.05	0.52^***^	0.49^***^	0.55^***^	—	
8. Future intention	6.44	0.91	0.04	0.09^*^	−0.07	0.45^***^	0.56^***^	0.59^***^	0.38^***^	—

Total *N* = 617, ^*^p < 0.05, ^**^p < 0.01, ^***^p < 0.001.

### Hypothesis testing

Table 2 shows results that were obtained after controlling gender, age, and education. As Table 2 (Equations 1 and 3) shows, relational job design positively predicted organizational commitment (*B* = 0.59, *SE* = 0.03, *p* < 0.01) and commitment to beneficiaries (*B* = 0.52, *SE* = 0.03, *p* < 0.01), supporting Hypotheses 1a and 1b. Table 2 (Equations 6) shows that organizational commitment positively predicted future volunteer intentions (*B* = 0.04, *SE* = 0.04, *p* < 0.01), supporting Hypothesis 2a. However, commitment to the beneficiaries did not predict future volunteer intentions (*B* = 0.48, *SE* = 0.04, *p* > 0.05), which does not support Hypothesis 2b. Table 2 (Equations 5) shows that relational job design positively predicted future volunteer intentions (*B* = 0.44, *SE* = 0.04, *p* < 0.01). The indirect effect of relational job design on future volunteer intentions through organizational commitment was significant (indirect effect = 0.28, SE = 0.40, 95% CI [0.21, 0.37]), supporting Hypothesis 3a. The indirect effect of relational job design on future volunteer intentions through commitment to the beneficiaries was not significant (indirect effect = 0.02, SE = 0.02, 95% CI [−0.03, 0.68]), which did not support Hypothesis 3b.

**Table 2:** Regression results for meditation effects and moderated meditation effects.

Predictors	Organizational commitment				Commitment to beneficiaries				Future intention			
	Equation 1		Equation 2		Equation 3		Equation 4		Equation 5		Equation 6	
	*B*	*SE*	*B*	*SE*	*B*	*SE*	*B*	*SE*	*B*	*SE*	*B*	*SE*
Gender	0.08	0.07	−0.03	0.06	0.03	0.07	0.00	0.07	0.12	0.08	0.08	0.07
Age	0.06	0.08	0.02	0.07	0.14	0.09	0.13	0.08	0.21^*^	0.09	0.18^*^	0.08
Education background	0.01	0.06	0.08	0.05	−0.01	0.07	0.01	0.06	−0.07	0.07	−0.08	0.62
Relational job design	0.59^***^	0.03	0.19^***^	0.04	0.52^***^	0.03	0.38^***^	0.05	0.44^***^	0.04	0.14^***^	0.04
Organizational commitment											0.48^***^	0.04
Commitment to beneficiaries											0.04	0.04
Perceived organizational support			0.61^***^	0.05			0.34^***^	0.05				
Relational job design × Perceived organizational support			0.03	0.02			0.10^***^	0.02				
*R^2^*	0.27		0.33		0.35		0.51		0.21		0.37	
*F*	57.47^***^		49.03^***^		81.84^***^		106.00^***^		41.46^***^		60.48^***^	

Total *N* = 617, ^*^p < 0.05, ^**^p < 0.01, ^***^p < 0.001.

Hypotheses 4a and 4b predicted that perceived organizational support would moderate the relationship between relational job design and organizational commitment, as well as job design and commitment to beneficiaries. In Table 2 (Equation 2), the interaction between relational job design and perceived organizational support did not predict organizational commitment (*B* = 0.03, *SE* = 0.02, *p* > 0.05), which did not support Hypothesis 4a. As shown in Table 2 (Equation 4), the interaction between relational job design and perceived organizational support positively predicted commitment to beneficiaries (*B* = 0.10, *SE* = 0.02, *p* < 0.01), supporting Hypothesis 4b.

**Figure 2:**
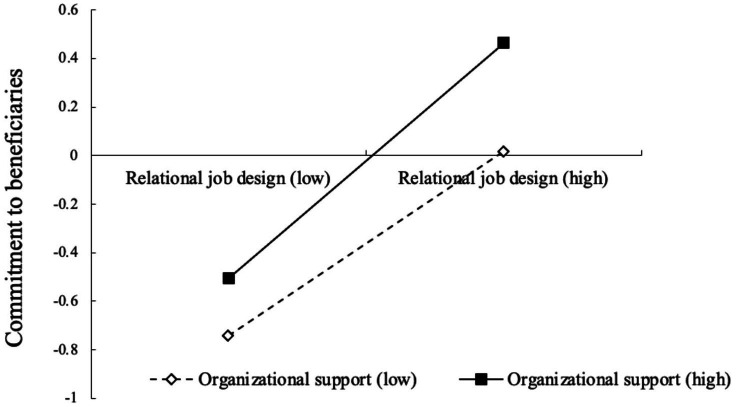
Perceived organizational support moderates the relation between relational job design and commitment to beneficiaries.

Further, simple slopes analysis suggested that the relationship between relational job design and commitment to beneficiaries was more positively significant for volunteers with high perceived organizational support (*B*_simple_ = 0.45, *p* < 0.001, 95% *CI* [0.35, 0.56]) than for volunteers with low perceived organizational support (*B*_simple_ = 0.28, *p* < 0.001, 95% *CI* [0.18, 0.37]). Figure 2 shows the interaction plot.

Hypotheses 5a and 5b predicted that perceived organizational support would moderate the indirect effects of relational job design on future volunteer intentions through organizational commitment and through commitment to beneficiaries. Because Hypothesis 4a was not supported, the moderated mediating effect through organizational commitment was not significant, either (effect = 0.01, boot *SE* = 0.02, 95% *CI* [−0.03, 0.04]; see Table 3), which does not support Hypothesis 5a. Results showed that the effect of moderated mediation was not significant (effect = 0.00, boot *SE* = 0.00, 95% *CI* [−0.01, 0.01]; see Table 3), which did not support Hypothesis 5b.

**Table 3:** Conditional indirect effects of relational job design on future intentions at different values of perceived organizational support.

	Perceived organizational support organizational support	Effect	SE_(boot)_	95%CI
	−1.03	0.08	0.03	[0.02, 0.15]
Organizational commitment	0.00	0.01	0.02	[−0.03, 0.04]
	0.73	0.71	0.10	[0.06, 0.16]
	−1.03	0.01	0.01	[−0.02, 0.04]
Commitment to beneficiaries	0.00	0.00	0.00	[−0.01, 0.01]
	0.73	0.02	0.02	[−0.03, 0.06]

## Discussion

Using Social Identity Theory (Tajfel and Turner, 1985; Turner, 1985) as a conceptual framework, we investigated the relationship between relational job design and future volunteer intentions, taking into account the effects of social factors such as perceived organizational commitment, commitment to beneficiaries, and perceived organizational support. The results showed that the association between relational job design and future volunteer intentions was mediated by organizational commitment, but not by commitment to beneficiaries. Relational job design was associated with higher commitment to beneficiaries (the first leg in one of the mediation processes), and this association was strengthened by perceived organizational support. Relational job design was also associated with higher organization commitment (the first leg in the other mediation process), but this relationship was not moderated by organizational support.

### Theoretical implications

The present study makes several theoretical contributions to the episodic volunteering literature. Previous studies have mainly focus on how individual difference factors and management practices might influence volunteers’ future intentions. The individual differences that have been studied include characteristics such as motivation and personal experience (Neufeind et al., 2013; Hyde et al., 2014; Cnaan et al., 2017). Management practices such as good communication, recognition, and rewarding job characteristics, have also been shown to be associated with higher future intentions (Taylor et al., 2006; Stirling et al., 2011).

The current results complement this earlier work by focusing on the importance of relational job design in terms of its association with episodic volunteers’ future intentions. Relational job design can be seen as a management practice that takes into account volunteers’ relatedness needs and their desire to have an impact on the organization and beneficiaries. Understanding the positive effects of relational job design can benefit episodic volunteers and also create a legacy of volunteering in the community, resulting in a win-win outcome (Kokolakakis et al., 2024). It has been shown that relational job design could improve ongoing volunteers’ attitudes and behavior concerning voluntary (Alfes et al., 2015), and our study found a similar pattern among episodic volunteers with regard to higher future intention.

We also documented the mediating role of organizational commitment in the association between relational job design and future volunteer intention. Consistent with Social Identity Theory (Tajfel and Turner, 1985; Turner, 1985), we would expect that volunteers develop a sense of self-worth as members of a volunteer group. This sense of self-worth will shape their identity as a member of the group and create an affective commitment to the group. Although some researchers hold the opinion that it is hard for episodic volunteers to develop organizational commitment because the organization is always temporary (Nichols, 2013; Hyde et al., 2016), our results suggest that being an episodic volunteer in mega sport events can foster organizational commitment.

Episodic volunteers at major sport events like the 7th CISM Military World Games are managed by governmental departments (including governmental sport departments and University Communist Youth League Committee) in China (Yao and Schwarz, 2017; Wu, 2018). Volunteers may identify with these volunteer organizations out of respect for the Chinese government. Their organizational commitment might reflect commitment to the government of China. Episodic volunteers’ commitment to the government may create a willingness to participate in a future event.

We also showed that relational job design was associated with higher volunteer commitment to beneficiaries. This finding complements similar results in research on ongoing volunteers (Valéau et al., 2013; Alfes et al., 2015; McAllum, 2017). However, our results showed that commitment to beneficiaries did not predict future volunteer intentions. Although volunteers form commitment to beneficiaries at one event, they might not identify beneficiaries in the present event as those in another one, because representative members participating in different events indeed varied a lot (Hyde et al., 2016; Wicker, 2017). As a result, it is reasonable to conclude that commitment to the beneficiaries volunteerism at the 7th CISM Military World Games cannot influence volunteer intentions to participate in another event.

Volunteers’ commitment to beneficiaries did not act as a mediator of the association between relational job design and future intention. However, relational job design was positively associated with commitment to beneficiaries (the first leg of the mediation process), and perceived organizational support moderated this association. Because of the characteristics of major sport events, episodic volunteers work at high intensity for a sustained period of time (Fairley et al., 2014; Cho et al., 2020). High work demands consume their resources to deal with challenges (Bakker and Demerouti, 2017). If volunteers have trouble connecting with beneficiaries because of the demands of the job, their commitment to them might weaken (Morrissette and Kisamore, 2020). By contrast, if perceived organizational support is high, providing episodic volunteers with resources to deal with demands, volunteers will overcome challenges and have a more positive experience. Their positive experience may increase their commitment to beneficiaries. In addition to being consistent with the Social Interaction Theory, this result is consistent with the job demands–resources (JD-R) theory (Demerouti et al., 2001), which holds that job resources can weaken the negative effect of job demands. Although commitment to beneficiaries failed to predict future intentions, it might still impact volunteer engagement and other motivation behavior (Munjal et al., 2021; Yoo et al., 2023).

Contrary to expectations, perceived organizational support did not strengthen the association between relational job design and organizational commitment. One aim of holding major sport events in China is to facilitate international relations by conveying a positive image to people all over the world (Yao and Schwarz, 2017; Gao et al., 2020). Unlike volunteers for other types of events, episodic volunteers in mega sport events might have a special motivation to show the strength of their country out of patriotism (Vetitnev et al., 2018; Ahmad et al., 2020). While connecting with beneficiaries, organizations might emphasize the importance of winning glory for the motherland, fulfilling volunteers’ patriotic motivation. No matter what hassles they face and how they perform during an event, episodic volunteers’ identification is easy to form once they have a chance to volunteer and have impact on others. As a result, organizational support might not be influential. Thus, regardless of whether the perceived organizational support is high or low, relational job design appears to not be associated with episodic volunteers’ greater organizational commitment design.

### Practical implications

The characteristics of volunteering have changed in the past decade (Wilson, 2012), and there has been an increase in episodic volunteering (Hustinx et al., 2008; Dunn et al., 2016). Our research provides new knowledge about episodic volunteers and has implications for increasing their intentions to continue volunteering. It will bring practical benefits for voluntary management, such as time savings, increasing volunteer motivation and satisfaction.

First, there was a positive association between relational job design and episodic volunteer future intentions. This result suggests that it may be beneficial to increase the relational job design of volunteers in major sport events. Supervisors in the volunteer organization can arrange more chances for volunteers to connect with beneficiaries and show volunteers that their work has an impact (Grant, 2007). Supervisors can give feedback to volunteers about what impacts they had on others, especially for volunteers do not have direct contact with beneficiaries cannot immediately see their impacts (Alfes et al., 2015). For instance, in mega sport events, some volunteers work away from athletes, umpires, and referees. Instead, they might stand guard outside the gymnasium or field or work for the executive committee. Supervisors could help those volunteers be aware of their importance for beneficiaries by reviewing volunteers’ contributions at daily short meetings, reading letters of thanks from beneficiaries, and arranging appearances by athletes. Moreover, some volunteers do not have a clear sense of their roles or how to identify their beneficiaries (Rogalsky et al., 2016). For example, volunteers whose task is to lead audience members to their seats might feel that they have no direct impact on athletes. Supervisors can help them to recognize that there are multiple beneficiaries, including spectators, helping them recognize their contributions as volunteers.

Second, organizational commitment was directly associated with volunteers’ intentions, suggesting the importance of improving episodic volunteers’ commitment to the larger group to which they belong. Supervisors could express their appreciation and respect for volunteers (Turner and Reynolds, 2001), for example by presenting them with certificates of honor, listening to their advice, and protecting their rights by creating official regulations. Clarifying and fulfilling volunteers’ motivations is also necessary for volunteers to recognize that their values are the same as those of their organization, potentially increasing organizational identification (Finegan, 2000; Cnaan et al., 2022). For example, when a volunteer’s motivation is to gain new skills (Bang et al., 2008), professional training can be arranged, which might also increase the success of volunteer activities.

Third, our study showed perceived organizational support strengthened the association between relational job design and commitment to beneficiaries. The results suggest that organizational support is important for episodic volunteers to develop commitment to beneficiaries. Although our results did not show that commitment to beneficiaries was associated with volunteers’ future intentions, other research has shown that commitment to beneficiaries improves volunteers’ attitude and performance when they volunteer again (Valéau et al., 2013; Alfes et al., 2015; McAllum, 2017). Supervisors can provide episodic volunteers with instrumental and emotional support (Mathieu et al., 2019), such as sharing experience, giving them good advice, and listening to and talking with them when they have a bad day.

## Limitations and directions for future research

We used a cross-sectional design, which precludes causal interpretations of the findings. Fortunately, we had a reasonable theoretical foundation to propose our hypotheses. Future studies could use a longitudinal design to fill this gap. Moreover, all measures were self-report, raising concern about common method bias (Podsakoff et al., 2003). However, common variance did not create undue bias in our data. Future researchers can consider collecting supervisor rating of organizational support and relational job design. In addition, in China, volunteers in major sport events are usually university students who do not have work experience (Wang and Wu, 2014; Jiang et al., 2017). Researchers in other countries could test this model among episodic volunteers who have working experience. In comparison to student volunteers, employee volunteers may have less time and energy to dedicate to volunteer activities, making it potentially more challenging to cultivate their commitment. Additionally, employee volunteers often possess distinct volunteer motivations compared to student volunteers (Rodell et al., 2015). This is particularly true for employees who have already fulfilled their relational needs or carry heavy workloads in the workplace; these individuals may prefer more humdrum and easy volunteer tasks as leisure activities. Future researchers should consider cultural factors, such as collectivism versus individualism. In collectivistic societies, volunteers may be more sensitive to organizational factors such as organizational commitment and support, which have been shown to significantly impact future volunteer intentions in our study. Conversely, in individualistic settings, individuals may focus more on personal factors like satisfaction and personality traits (Compion et al., 2022; Cho et al., 2023). As a result, it is essential for researchers to account for these differences when identifying potential mediators and moderators in volunteer research. These efforts can provide a test of the generalizability of our findings.

We assessed volunteers’ future intentions but not their actual behavior. Willingness is affected by many factors, and there could be an intention-behavior gap in volunteers’ future intentions (Sheeran and Webb, 2016). Our results also cannot indicate how long commitment lasts. If the commitment disappears before the next major event, volunteers might not have a strong motivation to help. A volunteer-registration system could track participation in subsequent events (Hu, 2020).

Contrary to expectations, perceived organizational support moderated the association between relational job design and organizational commitment, but not commitment to beneficiaries. Future studies can test the moderating effects of other organizational and individual factors. Informed by Social Identity Theory (Tajfel and Turner, 1985; Turner, 1985), several studies have shown that leadership, individual motivation, and personality traits like conscientiousness are important in interpreting the working environment and forming organizational identification (Van Knippenberg, 2000; Khan et al., 2019; Wang et al., 2021).

## Conclusion

We found that relational job design positively associated with future intentions of episodic volunteers in mega sport events through organizational commitment. Relational job design was also positively related to volunteers’ commitment to beneficiaries, and this relationship was significantly stronger when perceived organizational support was high. These results are in line with the Social Identity Theory, and have clear implications for designing volunteer jobs. A relational component could help make volunteers aware of their impact on others as a way to help them form organizational commitment, thus increasing their willingness to participate in another mega sport event in the future.

## Data availability statement

The raw data supporting the conclusions of this article will be made available by the authors, without undue reservation.

## Ethics statement

The studies involving humans were approved by Central China Normal University Ethics Committee. The studies were conducted in accordance with the local legislation and institutional requirements. The participants provided their written informed consent to participate in this study. Written informed consent was obtained from the individual(s) for the publication of any potentially identifiable images or data included in this article.

## Author contributions

JS: Conceptualization, Formal analysis, Investigation, Methodology, Writing – original draft, Writing – review & editing. HL: Formal analysis, Methodology, Resources, Writing – original draft. HM: Project administration, Supervision, Writing – review & editing.
